# Electrophysiological assessment of plant status outside a Faraday cage using supervised machine learning

**DOI:** 10.1038/s41598-019-53675-4

**Published:** 2019-11-19

**Authors:** Daniel Tran, Fabien Dutoit, Elena Najdenovska, Nigel Wallbridge, Carrol Plummer, Marco Mazza, Laura Elena Raileanu, Cédric Camps

**Affiliations:** 1 Institute for Plant Production Sciences, Agroscope, Route des Eterpys 18, CH-1964 Conthey, Switzerland; 2 University of Applied Sciences and Arts of Western Switzerland (HES-SO), Haute Ecole d’Ingénierie et de Gestion du Canton de Vaud (HEIG-VD), Route de Cheseaux 1, CH-1401 Yverdon-les-Bains, Switzerland; 3 Vivent SÁRL, Chemin de Varmey 1, CH-1299 Crans-près-Céligny, Switzerland; 4 University of Applied Sciences and Arts of Western Switzerland (HES-SO), Haute Ecole d’Ingénierie et d’Architecture Fribourg (HEIA-Fr), Bd de Pérolles 80, CH-1700 Fribourg, Switzerland

**Keywords:** Sensors and probes, Computational biology and bioinformatics, Plant sciences

## Abstract

Living organisms have evolved complex signaling networks to drive appropriate physiological processes in response to changing environmental conditions. Amongst them, electric signals are a universal method to rapidly transmit information. In animals, bioelectrical activity measurements in the heart or the brain provide information about health status. In plants, practical measurements of bioelectrical activity are in their infancy and transposition of technology used in human medicine could therefore, by analogy provide insight about the physiological status of plants. This paper reports on the development and testing of an innovative electrophysiological sensor that can be used in greenhouse production conditions, without a Faraday cage, enabling real-time electric signal measurements. The bioelectrical activity is modified in response to water stress conditions or to nycthemeral rhythm. Furthermore, the automatic classification of plant status using supervised machine learning allows detection of these physiological modifications. This sensor represents an efficient alternative agronomic tool at the service of producers for decision support or for taking preventive measures before initial visual symptoms of plant stress appear.

## Introduction

In 1873, following correspondence with Charles Darwin, electrical signals in plants were discovered by Burdon-Sanderson^1^ during a survey of Venus Flytrap (*Dionaea muscipula*). Based on this work, the remarkable Indian scientist, Bose^2^, at the beginning of the twentieth century, showed that plants generated electrical impulses in response to environmental stimuli similar to those of nerves in animals.

Plants have evolved several paths for long-range signal transmission between cells, tissues and organs in order to adapt their metabolism and development in response to a changing environment. This long-distance communication can be triggered by biotic or abiotic stimuli that are sensed locally by a few cells and translated into mobile signals such as small molecules, peptides, second messengers or phytohormones^3,4^. In contrast with these chemical signals, electrical signals are capable of transmitting information more rapidly over longer distances^3^. Electrical signals are known to regulate a wide variety of physiological processes. These functions may include growth, gas exchange, respiration, variation of photosynthesis and transpiration, and modification of gene expression (e.g. protease inhibitor)^5,6^.

Most studies on electrical signals in plants have been carried out under laboratory-controlled conditions. For instance, researchers focused on the description of standard signal characteristics e.g. amplitude, frequency, velocity, distance and direction of propagation^7–9^. However, studies are now dedicated to identifying the mechanisms of the signalling network, specifically the nature of the involved proteins, and understanding the interaction between the electrical, mechanical and chemical signalling mechanisms that form a complex signalling network that regulates physiological processes from the cellular level to the whole-plant level^10–12^. The approaches proposed to study electrical signalling include intracellular and extracellular measurements^13,14^. Intracellular measurements can directly record the value of an individual cell membrane potential, while extracellular measurements detect the total spatiotemporal sum of the depolarization–repolarization process in a large group of cells. Amongst them, in plants, there are three different types of electrical signals: action potentials (AP)^15^, variation potentials (VP)^16^, system potentials (SP) or electric potentials (EP)^17^. APs are induced by non-damaging stimuli (e.g. cold, mechanical and electrical stimuli), whereas VPs are induced by damaging stimuli (e.g. burning and cutting). Both of these are widespread signalling phenomena which can rapidly transmit information over long distances. EPs are a sub-threshold response induced by changes in environmental factors, e.g. soil moisture, water, fertility, light, air temperature and humidity^4,18^. Daily EP variations have been reported on different plant species such as maize, plum or avocado^19–21^ and strongly suggest a link with the nycthemeral/circadian rhythm. In *Populus trichoparpa* trees, Gilbert *et al*.^22^, reported a significant correlation between EP and sap flow. In water deficit conditions, EPs variations occur modifying the photosynthetic rates and stomatal conductance^23,24^.

For crop production in greenhouses, both climate management and control of irrigation are critical variables affecting yield and quality. Control of water uptake and the maintenance of water status are key for the survival and optimal growth of plants. Environmental factors such as radiation, air temperature, rainfall, and humidity have a high impact on plant water balances. Hence, plants require a coordinated and timely response in above-ground and below-ground organs to cope with the changing need to take up and preserve water. This is mediated by a complex signalling network, which includes, amongst others, electric signals. Hence, using electrical signals as the basis for a sensor to measure real-time plant water status is of great interest as an agronomic tool that enables continuous and non-destructive measurements to control irrigation.

However, both natural and man-made electromagnetic noise combined with low-voltage potential variations produced by plants, constrain researchers to the use of Faraday cages, due to the low signal to noise ratio. Therefore, most studies on electrical signals in plants have been carried out under laboratory-controlled or environmental-controlled conditions within a Faraday cage. Overcoming this constraint by improving the signal to noise ratio would enable recording of electrical signals from plants in all environments rather than just in laboratory conditions. The PhytlSigns device is a plant electrophysiological sensor that allows recording EP variations in “natural” conditions. In order to allow operation of the system outside a Faraday cage, several design features were implemented that improved the signal to noise ratio (See the Methods’ section).

A further challenge faced when investigating signalling networks in plants is the interpretation of the recorded signals. Often, mixed electrical potential waves are recorded in plants, for instance, as a result of overlapping between APs, VPs and EPs, creating a complex web of systemic information in which several electrical signals may be layered on top of each other in time and space^4^. Proper signal analysis in this case is complex and challenging^25^. Very recently Souza *et al*.^26^ proposed the concept of a “plant electrome” and showed that environmental stimuli could change some characteristics of the temporal dynamic of electrical signals. In animals, particularly humans, studies of the temporal dynamics of electric signals obtained by EEG (Electro-Encephalogram) have allowed the establishment of consistent relationships between an individual’s health state and complex EEG measures^27–30^. In plants, the exploration of the total bioelectrical activity has only recently emerged and is a growing area of interest. A step forward in this field will be to transfer the temporal-dynamic methods applied in animals and humans in order to obtain real-time monitoring of the physiological status of plants.

In this paper, we present the results of long-term recordings of bioelectrical activity of soilless cultivated tomatoes in a water deficit regimen, in a commercial greenhouse setting without use of a Faraday cage. In addition, we present preliminary results of the use of supervised machine learning techniques for identification and classification of plant water status using the acquired electrical signals.

## Results

### Improving the signal to noise ratio of electrical signals recordings outside of a Faraday cage

**Figure 1:**
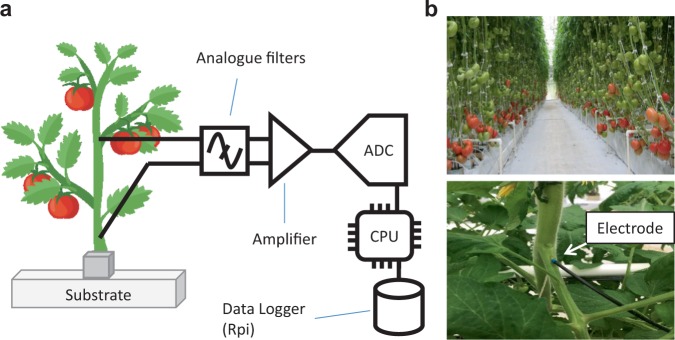
Enabling electrophysiological recordings outside a Faraday cage (**a**), Schematic representation of the PhytlSigns composed of an amplifier-voltmeter and analog to digital converter  are collected into a Raspberry Pi. (**b**), Experiments are performed on hydroponic tomato in soilless culture grown in greenhouse (*top*). The PhytlSigns device allows monitoring of electric signals in a ‘real’ environment without a Faraday cage.  An electrode is inserted in the tomato petiole at the top of the plant (*bottom*).

We utilized PhytlSigns devices to perform long-term recordings of EP variations on tomato in soilless culture in growing conditions similar to those used by commercial growers. The PhytlSigns device is composed of an amplifier that allows the measurement of voltage potential differences between plant tissues and a reference site (Fig. 1a). The active electrode is inserted into the petiole (in the vascular bundles) and the reference electrode in the substrate (Fig. 1b). This configuration allows long-term monitoring of EP variations since for commercial tomato production, standard practices includes different mechanical interventions (trellising, pruning, de-leafing or harvesting fruit). For long-term monitoring, the digitized data are recorded on a single board computer (Fig. 1a,b). Short-term or live recordings of electrical signals can be monitored with wireless devices (Supplementary movie S1). This instrumentation allows the recording of EP variations in “natural” conditions within a greenhouse without the use of Faraday cage.

### Long-term monitoring of electrical signals

**Figure 2:**
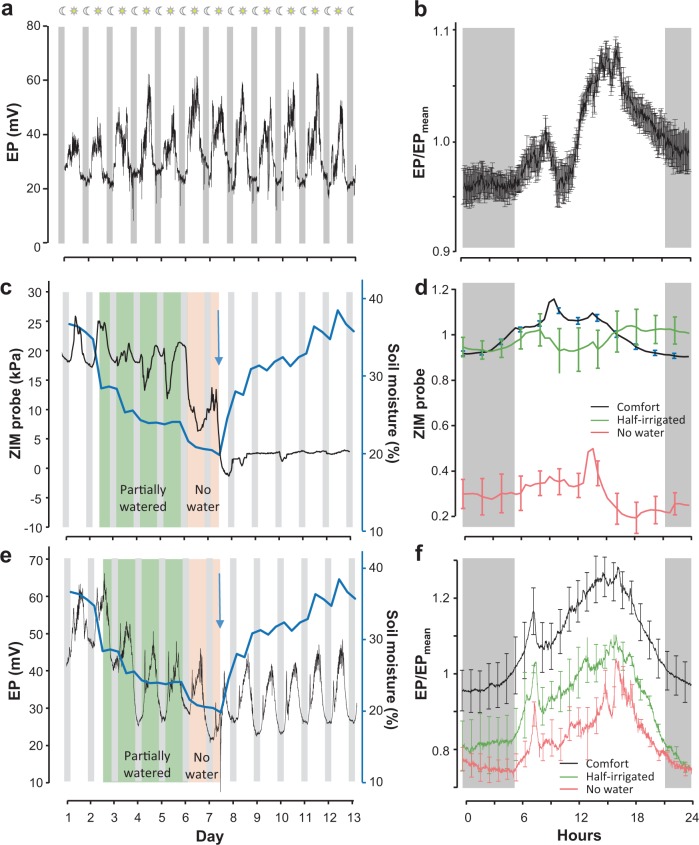
Electrical potential variations on tomato is modified in response to water deficit Hydroponic tomato plants in soilless culture are grown in the greenhouse. (**a**), Representative long-term recording of electric potential (EP) shows cyclic variations in controlled conditions. (**b**), EP variations from all tomato plants are split into 24 hour cycles and normalized. Results represent mean ± s.e.m, n = 60. Tomato plants were subjected to different irrigation regimens: optimal (*white*), half-irrigated during 4 days (*green*) or without irrigation for 36 hours (*red*). A comparison is done between commercialized (**c,d**), Yara-ZIM sensor (leaf turgor), and (**e,f**), PhytlSigns device (electrical signal) during these different irrigation regimens. Representative long-term monitoring of (**c**), ZIM probe showing leaf turgor and (**e**), EP variations. Evolution of water content in the substrate during the experiment is superimposed in blue with the secondary y axis. Blue arrow indicates the moment when roots were watered again after drought condition. The corresponding (**d**), ZIM and (**f**), PhytlSigns signals are normalized and averaged per 24-hours cycles in control (*black*), half-irrigated (*green*) and no water conditions (*red*). Results represent mean ± s.e.m (n ≥ 10).

Long-term (2 weeks) recordings of electrical signals of soilless tomato plants were obtained in growing conditions similar to those used by commercial growers (Fig. 1a,b). As a general observation, the electrical response shows a cyclic pattern with a minimum during late night/early morning and a maximum during the middle of the day (solar noon at about 14 h). To better characterize this daily rhythm and to overcome the variability of the basal electrical signal level, all signals were split into 24-hour cycles and normalized (Fig. 2b). In optimal growing conditions, an initial increase in voltage potential difference is observed at dawn lasting the first hours of the day. This is followed by a long-lasting voltage potential peak with a higher amplitude in the heat of the day (Fig. 2b), which coincided with high evapo-transpiration rate. The EP value is then reduced before sunset with a minimum during the night. It is noteworthy that the initial first peak was not observed systematically. These daily EP variations may reflect the daily plant activities with photosynthetic activity during light period whereas during night periods, only respiration remained. This is tightly linked to the sap and photosynthetic flow. These daily variations are similar (pattern and magnitude) to those recorded under controlled conditions on avocadoes or plum and strongly suggest a link with nycthemeral or circadian rhythm;^20,24,31^ however this needs further study.

For crop production in greenhouses, both climate management and control of irrigation are critical variables affecting crop yield and quality. We performed continuous EP monitoring of tomato plants during different water regimen conditions and explored whether variations in electrical signals corresponded to different plant stress levels. Plants were grown using optimal irrigation during the 2 first days, corresponding to substrate water content of 35 Vol%. Then the irrigation was reduced to 50% for 4 days leading to a drop of substrate water content maintained at around 25 Vol%. The following 36 hours, irrigation was completely stopped and the substrate water content diminished to 18 Vol%. (Fig. 2c,e; *secondary y axis*).

We undertook a comparison between a commercialized sensor ZIM (Yara plant technology) and the PhytlSigns signals (Vivent). The standard ZIM sensor detects leaf turgor^32^ reflecting a plant’s water status. In optimal growth conditions, ZIM sensor signals show a daily variation with a minimum leaf turgor during the night and a maximum reached during the day. During the water deficit regimen, the daily variation of leaf turgor is modified with a drop of turgor during the day. The more the substrate moisture diminishes, the more the leaf turgor drops (Fig. 2c-d) and the more the water potential increased (see Supplementary Fig. S1). In addition, tomato plants showed visible symptoms of water stress with leaf wilting after 24 hours (see Supplementary Fig. S1). This is consistent with a previous report showing the efficiency of leaf turgor sensors to detect water stress conditions^32^. However, under severe water deficit conditions, the signal from the ZIM sensor signal is lost and not recovered even when the irrigation is reinstated, showing a limitation of this sensor (Fig. 2c,d). Another limitation of ZIM sensors is that they can remain in place only for a short duration (less than 1 week) to avoid local physiological modifications (see Supplementary Fig. S2) and therefore, ZIM sensors need to be replaced periodically. The PhytlSigns voltage potential signal shows a progressive drop of the baseline during the half-irrigated regimen (Fig. 2e) with more extreme variations during the period of no irrigation. Both the duration and the magnitude of EP variations are significantly reduced with the diminution of the substrate moisture (Fig. 2f and see Supplementary Fig. S3). When reintroducing water to the roots (Fig. 2e, *blue arrows*), a transient downward spike in the EP was evoked and the substrate water content increased to 36 Vol% (Fig. 2e). Afterwards the EP variation slowly went back to initial basal level, showing the same daily rhythm, dark/light changes as prior to the water stress (Fig. 2e). These EP variations are consistent to those reported in avocado during water deficit conditions^20^. Our results demonstrate that in severe conditions of water deficit, monitoring the electrical variations is more effective than leaf turgor (ZIM sensor) during the recovery phase.

### Machine learning based classification of plant status: case study of water stress

We next investigated whether electrical signals provide information allowing an early detection, for example prior to visual symptoms being evident, of different plant states and could therefore help in the supervision of cultivated plants. In the first stage of data exploration, a principal component analysis (PCA) was performed on the raw data (electrical signals, mV) to evaluate the overall variability measured by the sensor and indirectly to evaluate its performance.

**Figure 3:**
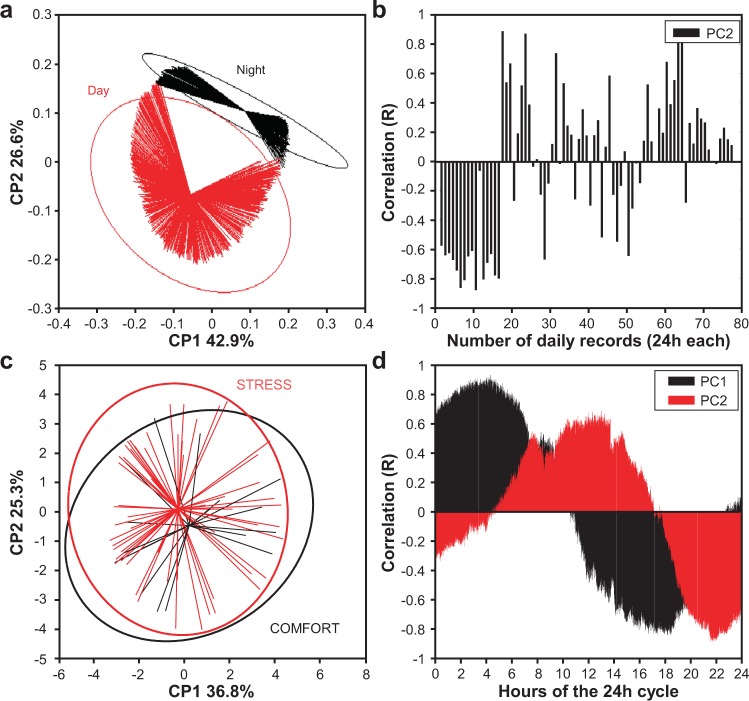
Electrical potential reflects nycthemeral rhythm Factorial map according to the first two factorial scores of the PCA performed on electrical signal data for (**a,b**), the day vs. night periods and (**c**,**d**), the water stress vs. comfort treatments. Red and blue ellipses (p = 0.05) highlight the variability of electrical signal.

This analysis makes it possible to describe, day after day, the variability of the electrical signal during a 24 h recording. Knowing the hours during which day or night is present, it is then possible to describe the daily variability of the electrical signal between the day and night phases. The factorial map of the PCA according to the first two factor scores (Fig. 3a), explains 69.6% of the variability, showing that the day and night periods can be separated into two distinct groups. This first result means that some portions of the recorded raw electrical signals contain relevant information to measure a different behaviour of the plant during the periods of day and night. To evaluate the weight of each daily record in the separation of the two groups (day vs. night), the correlation between the factorial coordinates of the second PC and the raw data of the electrical signals was calculated for each recorded day (Fig. 3b). As expected, it appears on reading the figure that most of the recording days of the electrical signal separate readily into one of the two groups. This result indicates that the variations of the electrical signal due to the day/night alternations are present in each day of recording and that they are therefore not due to chance but to one or several physiological phenomena.

We next analysed data to evaluate the water regimen (comfort or stress). The PCA map according to the first two principle components (PC), explains 62.1% of variability, allowing the data to be categorised into two different groups that overlap significantly (Fig. 3c). The correlation between the first two PC and the raw data has been calculated for each frequency of recording (Fig. 3d). The times of the day identified as relevant to separate the two groups (comfort vs. stress) were located near 4 am (PC1), 6 pm (PC1), between noon and 2 pm (PC2) and 10 pm (PC2). Despite these refinements, the analysis of the raw data does not clearly identify stressed and unstressed plants and further analysis of the signals using more sophisticated methods must be performed.

In order to better assess whether the acquired electrical signal is able to predict the plant status in different water regimen conditions, we also applied supervised machine learning techniques. We first test our approach on a clearly defined stimulus: light/dark. The electrical signals of the tomato plants monitored during a two-week period have been analysed as time series while different signal features were extracted (see Methods).

For predicting the daily rhythm, gradient boosted trees (GBT) showed the highest accuracy value (94.6%) among all of the tested models, followed by deep learning (DL) and logistic regression (LR) with 83.5% and 73.2%, respectively (Table 1). The GBT model yielded the highest precision and recall values in comparison with the other tested models (Table 1). The features that contribute the most in the modelling of these classifiers are shown in Supplementary Table S1. This approach confirms the PCA results and demonstrates that EP signal variations contain information about light or dark.

**Table 1:** Accuracy, Precision and Recall values for all prediction models to determine day or night.

Models	LR	DL	DT	RF	GBT
Accuracy (%)	73.2	83.5	62.0	61.4	94.6
Precision (%)	75.9	87.4	61.4	61.0	95.4
Recall (%)	81.2	84.8	99.6	99.8	95.6

For all model tested, GBT shows better performance. LR, logistic regression; DL, deep learning; DT, decision trees; RF, random forest; GBT, gradient boosted tree.

Then we test this approach on a more difficult defined state i.e. water stress. Since visual symptoms as well as the water potential increases appear significantly, 24 hours after the start of a water deficit regimen (see Supplementary Fig. S1), the plant drought state was defined according to the leaf turgor (Fig. 2b).

Similarly, for predicting the drought stress, GBT demonstrated better performance than other models in accuracy (98.5%), precision and recall value (Table 2). The two next best models were DL, with accuracy of 94.5%, and LR, with 83.6%. The Supplementary Table S2 give the list of the most important features used for modelling the classifiers to predict the water deficit stress. It is noteworthy that among all of the features extracted from the data, generalized Hurst exponents have a strong weight whatever the time window chosen.

**Table 2:** Accuracy, Precision and Recall values for all prediction models to determine water deficit.

Models	LR	DL	DT	RF	GBT
Accuracy (%)	83.6	94.7	78.5	76.2	98.5
Precision (%)	88.0	95.6	76.7	74.5	99.3
Recall(%)	88.4	96.8	99.2	99.8	98.5

For all model tested, GBT shows better performance. LR, logistic regression; DL, deep learning; DT, decision trees; RF, random forest; GBT, gradient boosted tree.

These results show that, through the use of advanced machine learning algorithms, the electrical signals acquired from plants have great potential in predicting both the daily rhythm and the water status in commercial tomato plants. Therefore, our findings support the idea that electrical signal measurements can help to adjust plant cultivation conditions in a proactive manner before visual symptoms appear due to drought. This is a significant finding. Other studies have explored strategies for the classification algorithms of low-voltage variations (microvolt)^33^ or raw non-stationary^34^ plant electrical signals reaching an accuracy of 84.4% and 73.7%, respectively. Further investigations are required to verify whether this model can be generalized to other plant species. It is noteworthy that this experiment was designed to analyse signals associated with daily rhythms and water stress and that the recorded signals do definitely contain information about other stressors like trellising, de-leafing, pruning, etc. With different labelling of the data the same recordings could be analysed for instance to look at pruning with the expectation that transient damage would be detected. Moreover, the acquisition of larger datasets is needed to refine the provided models and, additionally, to reliably select the most relevant features for discrimination of the different plant states, which would lead to an important reduction of the computing power needs.

## Conclusion

The results of this study show that, with both appropriate instrumentation and methods for data processing and analysis, it is possible to gauge, with a high-degree of confidence, plant water status using electrophysiological measurements in natural growing conditions i.e. without the use of a Faraday cage. We developed an electrophysiological sensor that allows continuous and stable long–term monitoring of plant electrical signals during several weeks without affecting plant physiology, that can be performed in commercial greenhouse for crop production. A case study on water deficit conditions showed an early change (downward) of the basal electrical signals leading to a modification of daily EP patterns in commercial tomato plants. This slight and slow modification that is hardly visible for producers, occurred within the plant allowing adaptation in order to cope with water deficit conditions until visual symptoms (i.e. leaf wilting) appear several hours or days later. This change is different from transient electrical variations observed in response to touch or injury^10,12,35,36^. Moreover, machine-learning based algorithms gave good classification performance confirming that plant electrical responses contain patterns that can be used for early identification of plant water status. Further detailed investigations are needed to better analyse water stress and the physiological modifications using different varieties and/or mutants to show that electrical signals are crucial for long distance drought signalling in plants. Current available sensors (i.e. porometer, Licor, Fluorpen, etc…) are designed to measure plant water status at a given time. These measurements must be made repeatedly if early detection of water stress is to be identified and this would be time consuming, inconvenient and expensive. Our findings support the idea that EP monitoring is a good indicator for water status and therefore could act as an aid in the supervision of plant cultivation. This represents, to our knowledge, the first potential application of plant electrical signal variations as an agronomic tool.

In greenhouse cultivation, maintenance and control of water status are key for optimal growth of plants and, in turn crop yields. Real-time assessment of plants’ physiological status would allow automatic irrigation management according to actual plant needs/demands and therefore diminish water waste. With population growth and rising affluence, smart water management is crucial for sustainable development, particularly in agriculture for food production. Further work, specifically, an extended dataset is needed to refine model algorithms in order to enhance the computational performance and to enable implementation in actual commercial greenhouse set-ups for irrigation management. Moreover, this opens new research avenues to study different growing conditions such as nutrients, pest or disease management that would in turn, allow automatic and real-time assessment of plant physiological status and behaviours. Development of these algorithms and associated sensors would be a useful tool to diagnose specific physiologic conditions, but also of great interest for producers in decision support and/or for taking preventive measures before initial visual symptoms of crop stress.

## Methods

### Plant material

Five tomato plants, variety Admiro (De Ruiter), grafted on Beaufort (De Ruiter) have been used in the present study plants from June to August 2018. Plants were grown in greenhouses at an Agroscope research station (Conthey, Switzerland) in either Rockwool mineral (Grodan, (ROCKWOOL B.V.), 6040 KD Roermond, The Netherlands) substrate or in an organic substrate composed of compost of bark (35%), a peat substitute (30%), Coco peat (20%) and topsoil 15% (Substrate 127, Ricoter, CH). An organic nutrient solution based on biogas digestate has been provided to plants as fertiliser.

### Plant turgor

Plant leaf turgidity has been monitored using the Yara Water-Sensor that measures the relative changes in the leaf’s turgor pressure of the plant (Yara International ASA

Drammensveien 131, 0277 Oslo - Norway). The pressure is expressed in kPa. Ten continuous records of plant leaves turgor were performed during 20 days each. At all, 200 night/day cycles of 24 h have been performed. The relative changes in turgor has been recorded with a stepwise of 1 minute.

### Climate monitoring

Climatic data close to the plants were monitored using the DGT-Senmatic climate computer in the greenhouse (Senmatic, Industrivej 8, 5471 Søndersø, Denmark). The air temperature (°C), air humidity (%), CO_2_ level (ppm), light level (Watt/m^2^), vapour pressure deficit (VPD) were recorded with a time step of 1 minute.

### Substrate monitoring

Temperature (°C), relative water content (%) and electro-conductivity (EC, mS.cm^-1^) in substrates were monitored daily using a WET sensor (WET-2, Delta-T Devices Ltd, Cambridge, UK). 3 measurements per day have been performed.

### Electrophysiology

Electrical signals produced by plants have been recorded using PhytlSigns devices from Vivent Sàrl (Crans-près-Celigny, Switzerland). Electrical potential was measured with custom-made electrodes. It consists in coaxial cable (2.79 mm diameter); the center conductor (silver coated copper filament diameter < 0.5 mm) wire was inserted into the petiole. In order to obtain a stable signal, the electrode should be inserted in the conducting bundles;^37^ thus, recording was checked during 72 hours following insertion and replaced if required. For each plant, we obtained a 2 weeks time series of data representing 77 daily cycles; the PhytlSigns device recorded the difference in electric potential between the substrate and a leaf petiole of the plant. The signal is sampled at 400 Hz with a gain of 4 and several filters are applied: low pass at 30 Hz and band-stop at 50 Hz and 100 Hz. The signal has been recorded in mV as a function of time. Digitized signal data were extracted and customized using Matlab software (Matlab, R2017a).

### PhytlSigns Bio Signals Data Acquisition System

The PhytlSigns device comprises an active electrode and a ground electrode both fabricated from 50 ohm impedance coax cable with an inner conductor of silver coated copper wire of diameter 0.5 mm. The outer conductor is a shielded copper braid with a waterproof jacket. Particular attention is paid to grounding throughout the instrument. The electrode is connected to a DC-coupled amplifier with appropriate filtering and noise cancellation followed by an analogue to digital signal converter and a data logger.

### Unsupervised descriptive analysis of raw signals

Prior to performing classification models using machine and deep learning, the raw data of electrical signals was analysed using principal component analyses to explore the variability of electrical signals with respect to day/night periods and water stress/control status.

Raw data of electrical signals were decimated at one point per minute for the different classes representing 1440 points for each 24 h cycles and 77 daily cycles from the five tomato plants representing. Analysis has been carried out for day/night periods, gathered in a matrix ${{\bf{X}}}_{{\rm{n}},{\rm{p}}}$ with *n* the number of frequencies of recording expressed in minutes (n = 1440) and *p* is the number of days monitored (p = 77). Analysis of water stress/control status has been carried out on a matrix ${{\bf{Z}}}_{{\rm{n}},{\rm{p}}}$ with *n* the number of days monitored and expressed as “stress” or “comfort” (p = 77) and *p* the number of frequencies of recording expressed in minutes (n = 1440).

### Data preprocessing

In order to prepare the raw data for the modeling of the classifiers, several preprocessing steps were applied: windowing, features extraction, normalization and labeling.

First, from the raw signal, in steps of 5 minutes we repetitively took seven samples of different window sizes, namely 15 s, 30 s, 1 min, 2 min, 5 min, 10 min and 30 min. Then, in each window, we extracted 26 features: simple statistical features (min, max, mean, variance, skewness, kurtosis and interquartile range), Hjorth parameters (mobility and complexity), Generalized Hurst exponent, Wavelet entropy (Shannon and logarithmic)^34^ and the estimation of the color of the noise (white, pink, brown, blue and purple)^33^. We also perform a wavelet decomposition, of order 1, 4 and 8, on the windowed signals and took the corresponding min, max and average value as additional features. In other words, this step allowed each 5 min of the time series to be described by 182 features in total.

To compensate for inter-plant variability in the extracted features, a normalization was also performed. More precisely, the values of each feature vector were transformed in the interval between 0 and 1 using the following formula:

$${x}_{nf=\frac{{x}_{f}-{x}_{f,min}}{{x}_{f,max}-{x}_{f,min}}}$$

where *x*_*f*_ and *x*_*nf*_ are the raw and the normalized feature vector respectively, while *x*_*f,min*_ the feature vector minimum and *x*_*f,max*_ its maximum.

To label the samples, we used the local ephemeris and the tables indicating the level of irrigation (100%, 20% and 0%) during the experiment. We consider that the plant is in drought stress 3 hours after irrigation is reduced or removed. Water stress status of the plants has been characterized and recorded by monitoring the turgor pressure of leaves (see Fig. 2c).

The 24,246 normalized and labeled samples compose the dataset we used for the supervised classification. The classes’ balance of our dataset is 14,621 (60.3%) day samples for 9,625 (39.7%) night samples and 7,467 drought-stressed (30.8%) for 16,779 normal (69.2%).

### Classification

We apply different supervised machine learning algorithms to model a classifier and evaluate its performance for the two classes independently (class 1: day/night and class 2: drought stressed/normal). The tested algorithms were the logistic regression (LR), the deep learning (DL), decision trees (DT), random forest (RF) and the gradient boosted tree (GBT). The dataset was split randomly into a learning set (80% of data) and a validation set (20%). Algorithms have been provided by H2O.ai which is an “Open Source Fast Scalable Machine Learning Platform” and XGBoost^38^ which is a scalable machine learning system for tree boosting.

## Supplementary information

Supplementary movie S1

Supplementary information
