# Supplementary material for: Electrophysiological assessment of plant status outside a Faraday cage using supervised machine learning
Source: Sci Rep. 2019 Nov 19;9:17073. doi: 10.1038/s41598-019-53675-4 (PMC6864072; doi:10.1038/s41598-019-53675-4)
Supplement: Supplementary file 2 — Supplementary information [file 41598_2019_53675_MOESM2_ESM.pdf]

Supplementary information for:

Electrophysiological assessment of plant status outside a Faraday cage using supervised machine learning

Daniel Tran[1, *], Fabien Dutoit[2], Elena Najdenovska[2], Nigel Wallbridge[3], Carrol Plummer[3], Marco Mazza[4], Laura Elena Raileanu[2] and Cédric Camps[1]

	Feature (window size)
1	Generalized Hurst exponent (30min)
2	Generalized Hurst exponent (10min)
3	Generalized Hurst exponent (5min)
4	Generalized Hurst exponent (1min)
5	Generalized Hurst exponent (15s)
6	Generalized Hurst exponent (30s)
7	Generalized Hurst exponent (2min)
8	Mean of the wavelet decomposition of order 4 (10min)
9	Brown noise (10min)
10	Skewness (10min)
11	Skewness (30min)
12	Mean of the wavelet decomposition of order 4 (1min)
13	Mean of the wavelet decomposition of order 4 (15s)
14	Mean of the wavelet decomposition of order 4 (30s)
15	Skewness (5min)
16	White noise (10min)
17	Pink noise (5min)
18	White noise (5min)
19	Maximum of the wavelet decomposition of order 1 (2min)
20	Pink noise (2min)

Supplementary Table S1 | The top 20 features that generally contributed the most in the modeling of the classifiers to predict day or night based on the electrical variations

	Feature (window size)
1	Generalized Hurst exponent (5min)
2	Generalized Hurst exponent (30min)
3	Generalized Hurst exponent (10min)
4	Generalized Hurst exponent (2min)
5	Generalized Hurst exponent (15s)
6	Generalized Hurst exponent (30s)
7	Generalized Hurst exponent (1min)
8	Maximum of the wavelet decomposition of order 1 (15s)
9	Maximum of the wavelet decomposition of order 1 (30s)
10	Maximum of the wavelet decomposition of order 1 (1min)
11	Minimum of the wavelet decomposition of order 1 (15s)
12	Minimum of the wavelet decomposition of order 1 (30s)
13	Minimum of the wavelet decomposition of order 1 (1min)
14	Minimum of the wavelet decomposition of order 1 (5min)
15	Minimum of the wavelet decomposition of order 1 (2min)
16	Minimum of the wavelet decomposition of order 1 (10min)
17	Interquartile range (15s)
18	Interquartile range (30s)
19	Interquartile range (1min)
20	Maximum of the wavelet decomposition of order 1 (2min)

Supplementary Table S2 | The top 20 features that generally contributed the most in the modeling of the classifiers to predict water-deficit stress based on the electrical variations

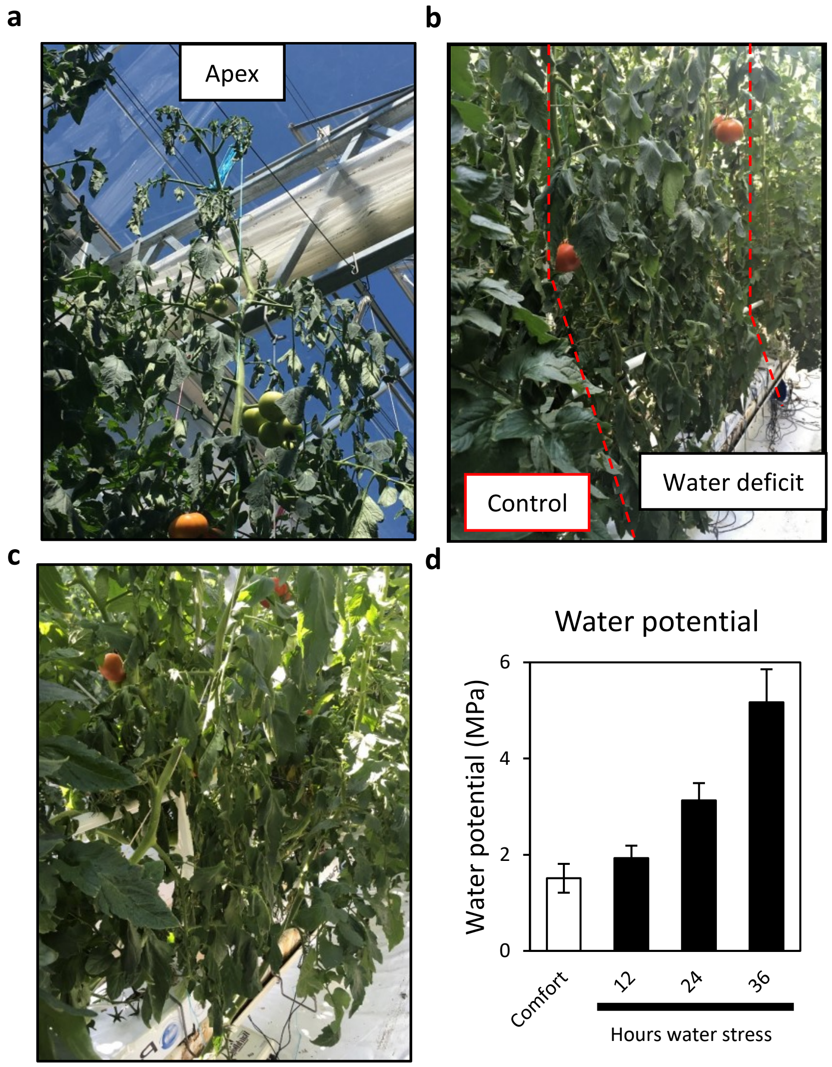

Supplementary Figure S1 | Hydroponic tomato plant in soilless culture shows early wilting at **a,** the apex and **b-c,** the whole plant, 24 and 36 hours, following water deficit conditions respectively. **d,** Evolution of water potential in leaves in comfort condition and the following hours after water stress deficit. Results are mean ± s.e.m, n=6.

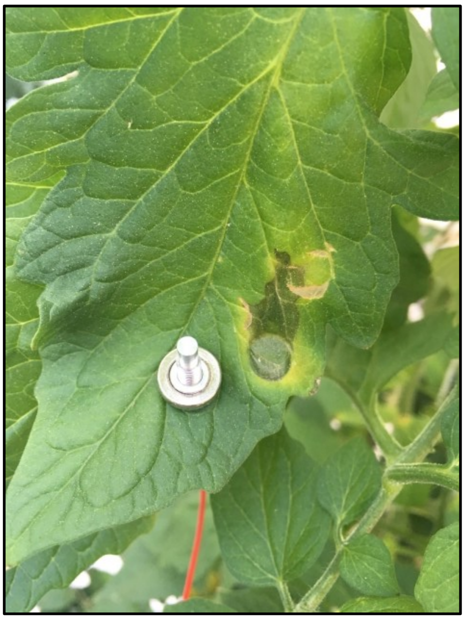

Supplementary Figure S2 | ZIM sensors place on the leaf induce local necrosis after 7 days use.

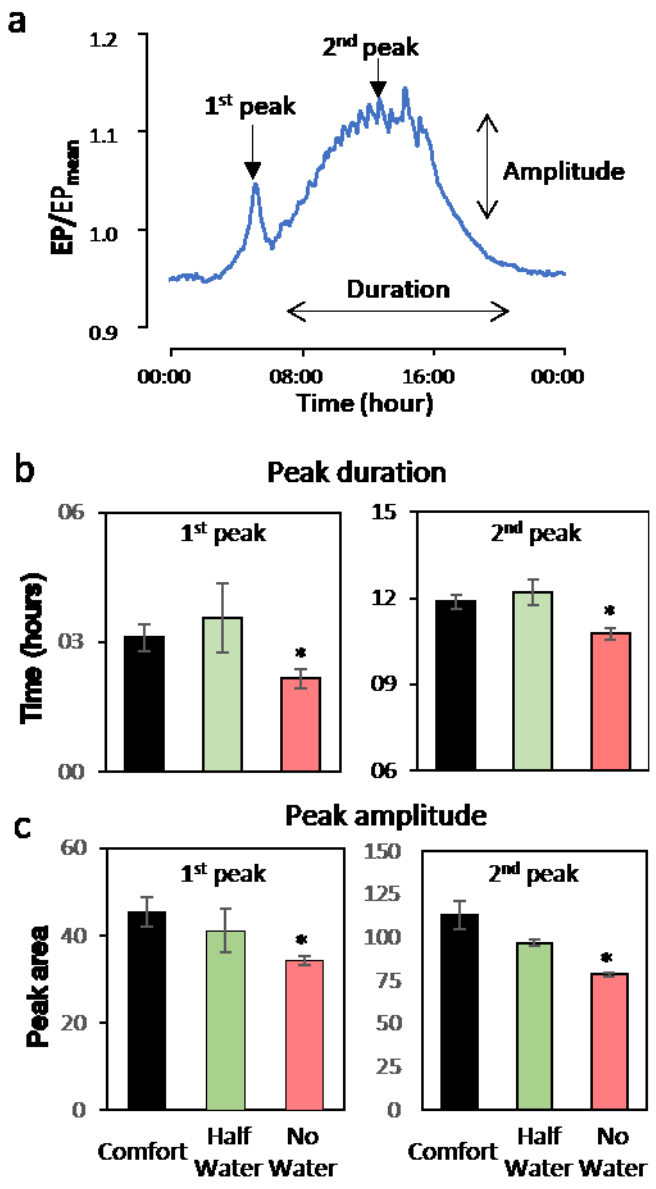

Supplementary Figure S3 | a, Typical signature of normalized electrical potential (EP) variations for hydroponic tomato plant in soilless culture. The EP shows a first peak in the morning followed by a long-lasting one in the afternoon. In response to water deficit, **b,** the both two peaks duration are affected, as well as **c,** the amplitude.

Results are mean ± s.e.m, n ≥ 10. Asterisks indicates significant difference from plants in comfort conditions (t-test, $p<0.05$).

Supplementary Video S1 | Live recording of electrical signal outside Faraday cage on hydroponically tomatoes in soilless culture
